# Ice, Ice, Maybe? Legionella-based Improvements in Environmental Patient Safety

**DOI:** 10.1017/ash.2025.202

**Published:** 2025-09-24

**Authors:** Carrigan Hayes, Antonisha Seay, Ioana Chirca, Leigh Ann Kelly, Delrico De Guzman

**Affiliations:** 1 AdventHealth Orlando; 2 Adventhealth

## Abstract

**Background:** Following the identification of Legionella pneumophilia by next generation sequencing (NGS) testing in an immunocompromised patient in June 2024, an extensive environmental sampling initiative was launched to determine possible contamination sources. **Method:** The water management team was immediately notified and performed initial testing on the unit where the positive case was identified, in which six ice and water machines, two showers, and four sinks were all sampled from two inpatient oncology units. **Result:** Only 4 of the 270 identified ice machines were tested every quarter. Since there was no randomization in the testing, this did not provide an accurate representation of the effectiveness of the hospital’s water management system. The results identified an ice machine located in a low traffic area of the unit which resulted positive for Legionella pneumophilia. **Conclusion:** Our findings led to the identification of the implicated ice machine in a low traffic area of the unit as our primary source, highlighting the need for more robust water monitoring practices across high-risk areas. When interviewing the nursing team, it was mentioned that the patient frequently requested ice chips to consume due to oral lesions, and nursing flow confirmed the impacted ice machine was the source of the patient’s ice chips. In response, we expanded water testing protocols from 4 (1%) to 22 (8%) randomized ice machines in quarter three testing, resulting in the identification of 10 of the 22 machines testing positive for Legionella, a 45.5% positivity rate. This allowed us to change our water testing program moving forward to include 120% more ice machines in the quarterly testing program. We also implemented a phased approach to address the low flow machines throughout the hospital that are at risk of bacterial production in which we are gradually installing flow meters and monitoring usage for approximately one to two months per machine. Machines that are deemed low flow will be removed from the unit. We aim to reduce low flow ice machines on the inpatient units by 50% by the end of quarter 4 in 2025. These changes have reinforced our commitment to patient safety by limiting pathogen exposure and operating from an infection prevention standpoint. This investigation underscores the critical role of environmental monitoring and ongoing assessment of water-related equipment in healthcare settings.

